# Brain Responses to a Self-Compassion Induction in Trauma Survivors With and Without Post-traumatic Stress Disorder

**DOI:** 10.3389/fpsyg.2022.765602

**Published:** 2022-03-22

**Authors:** Jennifer L. Creaser, Joanne Storr, Anke Karl

**Affiliations:** ^1^Department of Mathematics, University of Exeter, Exeter, United Kingdom; ^2^Department of Psychology, University of Exeter, Exeter, United Kingdom

**Keywords:** post-traumatic stress disorder, subthreshold PTSD, self-compassion induction, alpha asymmetry, EEG microstates

## Abstract

Self-compassion (SC) is a mechanism of symptom improvement in post-traumatic stress disorder (PTSD), however, the underlying neurobiological processes are not well understood. High levels of self-compassion are associated with reduced activation of the threat response system. Physiological threat responses to trauma reminders and increased arousal are key symptoms which are maintained by negative appraisals of the self and self-blame. Moreover, PTSD has been consistently associated with functional changes implicated in the brain’s saliency and the default mode networks. In this paper, we explore how trauma exposed individuals respond to a validated self-compassion exercise. We distinguish three groups using the PTSD checklist; those with full PTSD, those without PTSD, and those with subsyndromal PTSD. Subsyndromal PTSD is a clinically relevant subgroup in which individuals meet the criteria for reexperiencing along with one of either avoidance or hyperarousal. We use electroencephalography (EEG) alpha-asymmetry and EEG microstate analysis to characterize brain activity time series during the self-compassion exercise in the three groups. We contextualize our results with concurrently recorded autonomic measures of physiological arousal (heart rate and skin conductance), parasympathetic activation (heart rate variability) and self-reported changes in state mood and self-perception. We find that in all three groups directing self-compassion toward oneself activates the negative self and elicits a threat response during the SC exercise and that individuals with subsyndromal PTSD who have high levels of hyperarousal have the highest threat response. We find impaired activation of the EEG microstate associated with the saliency, attention and self-referential processing brain networks, distinguishes the three PTSD groups. Our findings provide evidence for potential neural biomarkers for quantitatively differentiating PTSD subgroups.

## Introduction

Posttraumatic stress disorder (PTSD) is a detrimental mental health condition diagnosed after psychological trauma such as exposure to actual or threatened death, serious injury, or sexual violence. The Diagnostic and Statistical Manual of Mental Disorders (DSM-5) characterizes PTSD by persistent intrusive memories, altered arousal, avoidance and negative alterations in mood that result in significant distress and social and occupational dysfunction (American Psychiatric Association [APA], 2013). PTSD is a global affliction with a cross-national lifetime prevalence of 3.9% and amongst those exposed to trauma 5.6% (Koenen et al., 2017). There is an increasing body of literature on the higher prevalence of PTSD in COVID-19 survivors (Kaseda and Levine, 2020) and healthcare professionals working during the pandemic (D’ettorre et al., 2021).

There is increasing evidence that although PTSD symptoms may sit below the diagnostic threshold, patients can still be highly symptomatic (Bradley et al., 2005). A category of subsyndromal (or partial) PTSD has been defined, in which individuals meet the criteria for reexperiencing along with one of either avoidance/numbing or hyperarousal but remain below the diagnostic threshold (Hickling and Blanchard, 1992; Blanchard et al., 1996). Subsyndromal PTSD is a clinically significant subcategory of PTSD that has been associated with substantial disability and elevated suicide risk (Marshall et al., 2001). However, there is a lack of reliable neurological biomarkers to distinguish PTSD subtypes and symptom patterns in the clinic.

Recent developments in this direction have come from studying the underlying neural mechanisms and corresponding pathophysiology of PTSD (Rabe et al., 2006). Studies of brain activity and emotion are guided by two related theoretical neuropsychological frameworks. Davidson proposed the approach-withdrawal model relating anterior asymmetry with affective style (Davidson, 1992). The valence-arousal model of Heller expanded on the approach-withdrawal model by including posterior asymmetry (Heller, 1993). The avoidance and numbing symptomology of PTSD and subthreshold PTSD can result from overactivation of the withdrawal system, which in Davidson’s model is associated with right anterior activation. Hyperarousal aligns with Heller’s hypothesizes that arousal manifests as hyper-activity in the right parieto-temporal region (Heller, 1993; Heller and Nitschke, 1998). Moreover, people with PTSD have high levels of trait anxious apprehension, which has been associated with an anxious arousal response measured by the increase in right parietal activity, in response to emotional narrative (Heller et al., 1997).

Evidence for these models and their link to PTSD symptoms comes from the study of electroencephalography (EEG) alpha-band (8–13 Hz) power. EEG alpha-band activity is considered to be inversely proportional to mental effort (Davidson, 1988). A common measure is alpha asymmetry that quantifies the difference in power between the left and right hemispheres in the anterior and posterior regions (Reznik and Allen, 2018). Parietal alpha asymmetry is associated with arousal and has been consistently linked with PTSD diagnoses and symptomology (Butt et al., 2019). Level of right parieto-temporal activation has been shown to discriminate PTSD from major depressive disorder (Metzger et al., 2004; Kemp et al., 2010). Right anterior and posterior activation was identified in response to trauma related pictures prior to trauma-focused cognitive behavior therapy (CBT) (Rabe et al., 2006) but no longer after successful PTSD treatment (Rabe et al., 2008). Increased left anterior alpha band activity was observed in response to olfactory stimulation (McCaffrey et al., 1993). One study on EEG alpha asymmetry for subthreshold PTSD identifies the same pattern of right anterior and posterior activation (Rabe et al., 2006). Using trauma exposed controls, Meyer et al. (2016) found that individuals without PTSD have significantly higher left-sided activity in response to negative pictures compared to those with PTSD. These studies may indicate that the pattern of greater right than left activation which also responds to psychological treatment is a promising clinical marker of PTSD symptoms (Meyer et al., 2016; Butt et al., 2019). However, a myriad of results from alpha asymmetry studies in major depressive disorder have shown inconsistent results and low retest reliability (Debener et al., 2000; Reznik and Allen, 2018).

More contemporary models consider brain activity as the result of switching between interconnected subnetworks of brain regions (Breakspear, 2017). This subnetwork approach is better for understanding mental health conditions because advances in neuroimaging have revealed multiple bilateral brain regions are active at once, indicating a network structure, and there are fast transitions (on the order of milliseconds) between networks. There has been a corresponding shift toward framing PTSD as a network based neurological disorder, using models of abnormal connectivity in the salience, central executive and default mode networks (Koch et al., 2016; Akiki et al., 2017). These networks have been directly related to distinct EEG topographic maps called *EEG microstates* (Britz et al., 2010; Yuan et al., 2012; Milz et al., 2016). Microstate analysis is an increasingly popular tool, where the EEG is broken down into a small number of dominant spatio-temporal EEG topographies. These topographies are compared to the original EEG and the one with the highest correlation at each time point is assigned on a winner-takes-all basis. This technique reduces the dimension of each individual’s EEG recording to a single microstate sequence. The number of dominant microstate maps identified in the majority of studies is four (Khanna et al., 2015; Michel and Koenig, 2018). These four classic maps have been found to be consistent across the lifespan (Tomescu et al., 2018) during wakeful rest and sleep (Brodbeck et al., 2012) and have been shown to capture between 60 and 80% of the variance of the data (Khanna et al., 2015). In joint EEG-fMRI studies these four classic maps have been associated with the auditory, visual, salience and attention networks (Britz et al., 2010; Yuan et al., 2012). These maps have also been used to identify patterns of activation in the default mode network during meditation (Panda et al., 2016).

The temporal dynamics of microstate sequences have been shown to be a powerful tool to distinguish neurological and psychiatric disorders (Khanna et al., 2015; Michel and Koenig, 2018). Microstate analysis has been used to differentiate low and high arousal levels (Gianotti et al., 2008), study emotion regulation (Al-Fahad and Yeasin, 2019; Zerna et al., 2021), and shown to differentiate individuals with mood and anxiety disorders from controls in resting state EEG (Al Zoubi et al., 2019). Joint EEG-fMRI study of resting state dynamics in combat exposed veterans with and without PTSD identified a microstate that was associated with the dorsal default model network (Yuan et al., 2018). They found that the occurrence rate of this microstate correlated with the PTSD Checklist (PCL) score where higher occurrence indicates higher PCL score and could be used to distinguish the two groups. This indicates the potential for EEG microstate analysis to stratify groups with different PTSD symptom severity, and to detect treatment response.

Existing trauma-focused therapies such as trauma-focused CBT and eye movement desensitization and reprocessing (EMDR) provide clinically meaningful symptom reduction (Watts et al., 2013). However, up to two-thirds of participants maintain their diagnosis of PTSD and EMDR and CBT have high attrition rates (10–20%) related to concerns that exposure based therapies will exacerbate symptoms (Zayfert et al., 2005; Schottenbauer et al., 2008; Steenkamp et al., 2015). The high attrition rates of trauma-focused therapies have necessitated the development of a range of complementary treatments (Boyd et al., 2017). These treatments are designed to sit alongside trauma-focused therapy for those who are not able to benefit from trauma focused therapy alone. Mindfulness based interventions in particular show significant symptom reduction, low dropout rates, and have been shown to reduce avoidant behavior in PTSD (Kimbrough et al., 2010; King et al., 2013).

One of the key mechanisms identified in cognitive models of the development and maintenance of PTSD is self-criticism and negative-self (Ehlers and Clark, 2000; Thompson and Waltz, 2008). Excessive negative-self can generate a sense of threat that leads to overactivation of the withdrawal system that in turn negatively impacts on successful trauma processing and hence can affect engagement with existing trauma-focused psychological treatment (Ehlers and Clark, 2000; Marx and Sloan, 2005). Self-compassion directly addresses the negative self-mechanism and has been found to be a driver of change in PTSD symptoms (Thompson and Waltz, 2008; Kearney, 2013). Self-compassion is defined as being kind to oneself in instances of pain or failure; perceiving one’s experience in the context of wider human experience; and being mindful of painful thoughts or feelings without overidentifying with them (Neff, 2003a). Thomson and Waltz identified a significant correlation between avoidance symptoms in PTSD and levels of self compassion where higher self-compassion correlated with low avoidance (Thompson and Waltz, 2008). A pilot study in veterans for the effect of loving kindness meditation (LKM) for PTSD showed that higher levels of self-compassion cultivated through a 12-week LKM are associated with reduced PTSD symptoms (Kearney, 2013). These two studies indicate that, used alongside trauma focused-therapy, increasing levels of self compassion could help lower experiential avoidance and lower treatment dropout rates. Although compassion is not an active element of present centered therapies, such as mindfulness based cognitive behavioral therapy (MBCT), they have been shown to increase dispositional self-compassion (Kuyken et al., 2010).

Cultivation of self-compassion is the focus of loving kindness meditation (LKM). The research on LKM and self-compassion is still in its infancy. Kirschner et al. (2019) developed a short LKM for the Self (LKM-S) intervention to study the cultivation of self-compassion in a healthy population. The authors found significant physiological changes in the response to the LKM-S including increased heart rate variability, a marker of adaptive emotion regulation, and decreased heart rate and skin conduction levels, markers of decreased physiological arousal (Kirschner et al., 2019). These physiological changes were accompanied by self-reported increases in self-compassion and reductions in self-criticism. In contrast, in a follow up study, individuals with major depressive disorder and higher levels of self-reported self-criticism showed an adverse physical arousal response to the LKM-S indicated by significantly elevated skin conductance levels (Kirschner et al., 2022). They conclude that the LKM-S taps into automatic self-referential processing and the level of stress response can be seen as a physiological marker for negative-self bias. They do not consider the underlying neural correlates of this paradigm, but note that this would be useful for further understanding.

The response to the LKM-S intervention can be regarded as a physiological marker for negative-self bias. The LKM-S intervention, although compassion is not an active element, targets similar mechanisms as MBCT. These are physiological arousal reduction and increase of parasympathetic activation (Kirschner et al., 2019) as indicators of reduced threat and improved emotion regulation (Thayer and Lane, 2000) and negative-self and self-criticism (Kirschner et al., 2019, 2022) and hence relevant for some key PTSD maintenance mechanisms. The negative-self is particularly important given the hypothesized role of a disturbance in self-organization which has been defined as a key criterion of a subgroup of trauma survivors recently classified as complex PTSD (Brewin et al., 2017) who also tend to benefit less well and drop out more often from recommended trauma-focused treatments (Cloitre, 2015). For these individuals, self-compassion has been proposed as a meaningful treatment target (Karatzias et al., 2019). To our knowledge there have not been any trials looking at the neurological and physiological correlates of the response to the LKM-S in individuals with PTSD. Here we explore its suitability for differentiating different levels of PTSD.

In this paper, we focus on civilian trauma survivors with varying PTSD symptom levels and categorize them into non, subthreshold and full PTSD groups based on their symptomology. We measure the neurophysiological (EEG) and physiological [electrocardiogram (ECG) for heart rate and heart rate variability, and skin conductance] reactions during the LKM-S self-compassion induction as described in Kirschner et al. (2019, 2022). We also measure self-reported state mood and self-perception before and after the LKM-S induction. We hypothesize a higher threat response in the PTSD groups in reaction to the shift of focus to direct loving kindness to themselves, as measured by increased skin conductance level (SCL) and decreased heart rate variability (HRV) in line with the aberrant physiological responses observed in Kirschner et al. (2022). Moreover, the levels of hyperarousal, and therefore the levels of threat response, may be higher in subthreshold PTSD (Hickling and Blanchard, 1992; Blanchard et al., 1996) allowing further differentiation of the subthreshold PTSD group.

We investigate the relationship between neural activation and autonomic response patterns to the LKM-S self-compassion induction. The neural correlates of the overactivation of the withdrawal system in PTSD generated by excessive negative-self is associated with right anterior activation and posterior asymmetry in the cognitive models by Heller and Davidson (Davidson, 1992; Heller, 1993; Heller and Nitschke, 1998). We hypothesize that the magnitude of the parietal alpha asymmetry will distinguish between the groups. Specifically, that the sPTSD will have greater right than left activation than the other two groups due to the higher levels of hyperarousal and lower levels of numbing.

Finally, we explore the temporal properties of the underlying neural networks via EEG microstate analysis during the LKM-S induction. Due to large-scale functional connectivity dysfunction of the underlying brain networks in PTSD (Koch et al., 2016; Akiki et al., 2017) we expect alterations in the microstate statistics during the processing of the LKM. Our exploratory hypothesis is that we expect to see alterations in the temporal dynamics of the microstate sequences for the microstate maps that correspond to the executive network that controls cognitive and emotional regulation, the saliency network that biases the threat response, and the default mode network that activates under self-referential processing. This study will contribute to the understanding of the neurobiological mechanisms underlying mindfulness and loving kindness-based approaches which are currently in their infancy.

## Materials and Methods

### Design and Participants

We used a mixed experimental design assessing within-subject (pre and post LKM-S and LKM-S focus on self or others) and between-subjects (nPTSD, sPTSD, and fPTSD groups) effects on self-reported measures of self-compassion, alpha asymmetry, and psychophysiological responses. Ethical approval for this study was granted by the University of Exeter, School of Psychology Ethics Committee, local trusts, and the National Health Service National Research Ethics Service (14/SW/0055). Additionally, all experimental procedures were developed in collaboration with the Lived Experience Group at the Mood Disorders Centre.

Participants were recruited from two mental health trusts, GP surgeries, and the University of Exeter. Recruitment was based on an opt-in system, where participants contacted the researcher after seeing posters, leaflets, or advertisements. A total of 68 participants were telephone screened after showing interest in the study. Participants were included if they were over 18 years old and had a history of trauma that happened at least 6 months prior to study participation and met PTSD criterion A of the DSM-IV (The American Psychiatric Association [APA], 1994). Participants were excluded when they reported (a) history of schizophrenia, another psychotic disorder, or bipolar disorder; (b) trauma that happened less than 6 months ago; (c) change of psychiatric medications treatment within the last 3 months; (d) currently taking cardiovascular medication; (e) history of cardiac problems or (f) history of neurological conditions known to affect EEG measurements such as epilepsy or brain surgery.

### Self-Report Measures

*Visual Analog scales* (VAS) were used to assess the effect of the loving kindness induction on individuals state self-compassion and state self-criticism (scores ranging from 0 to 100); the full prompt can be found in Kirschner et al. (2019). State self-compassion was assessed by two questions (published Cronbach’s α = 0.73) adapted from the SCS (Neff, 2003b), state self-criticism was based on the FSCRS (Gilbert et al., 2004).

*The PTSD Checklist Civilian version* (PCL-C; Blanchard et al., 1996) was administered after the experiment to assess current PTSD symptom severity (scores between 17 and 85) and status according to DSM-IV criteria. The PCL-C is a validated self-report rating scale for PTSD comprising of 17 items that correspond to the key symptoms of PTSD on a five-point Likert scale. The PCL-C has excellent psychometric properties (published Cronbach’s α = 0.94–0.97; current study α = 0.91). Self-report assessment of PTSD was chosen over structured interviewing due to time and funding restrictions. However, the PCL-C has been shown to be highly correlated (*r* = 0.93) with the Clinician Administered PTSD Scale (CAPS; Blake et al., 1995), the gold standard assessment for PTSD.

The PTSD is characterized (conservatively) by PCL-C scores greater than 44 (Blanchard et al., 1996). Questions on the PCL-C are split into three categories: intrusions, avoidance/numbing, and hyperarousal. A question score above 3 satisfies the diagnostic criteria for the symptom to be present. The criteria for full PTSD (fPTSD) are at least one intrusion symptom, 3 avoidance and numbing symptoms and at least 2 hyperarousal symptoms, and a total PCL score > 44. The criteria for subthreshold PTSD (sPTSD) are at least one intrusion symptom, with either 3 avoidance and numbing symptoms or at least 2 hyperarousal symptoms. The no PTSD group (nPTSD) does not meet the criteria for any symptom group and has a PCL score below 44.

*The Self-Compassion Scale Short form* (SCS-SF) an abbreviated version of the Self-Compassion Scale (Neff, 2003b), was used to assess trait level self-compassion where higher scores indicate a greater amount of self-compassion (scores ranging from 0 to 60) (Raes et al., 2011). It is a 12-item self-report for participants to measure how they typically act toward themselves during challenging times on a five-point Likert scale. The SCS-SF has good psychometric properties (Published Cronbach’s α = 0.86; Current study α = 0.89–0.91). Comparison between groups was performed using paired t-tests with Bonferroni correction.

*The Depression, Anxiety and Stress Scales* (DASS) was used to assess levels of comorbid anxiety, depression and stress (Lovibond and Lovibond, 1995). The DASS is a 42-item self-report questionnaire which uses a four-point Likert scale. It includes three subscales designed to measure levels of depression, anxiety and stress. The total score in each sub scale is reported for each participant. The DASS has been shown to have good psychometric properties (Brown et al., 1997) (published Cronbach’s α = 0.89–0.96; Current study α = 0.92–0.96). Comparison between groups for each subscale was performed using paired t-tests with Bonferroni correction.

*The Forms of Self-Criticism/Self-Reassuring Scale* (FSCSR) was used to assess self-criticism levels (Gilbert et al., 2004). This is a 22-item scale which assesses participants’ thoughts and feelings about themselves during a perceived failure using a 5-point Likert scale. Two subscales measure forms of self-criticizing and one subscale measures tendencies to be reassuring to oneself. The mean score in each subscale was recorded for each participant. Published Cronbach’s α = 0.86–90; Current study α = 0.74–0.95 (Inadequate self = 0.93, Reassure self −0.88, Hated self = 0.89). Comparison between groups for each subscale was performed using paired t-tests with Bonferroni correction.

### Recording Devices

#### Electroencephalogram

Electroencephalogram (EEG) was acquired using 64 active Ag/AgCl electrode embedded in a cap (ActiCap, Brain Products, Munich, Germany), connected to EEG amplifiers (Brain Amp, Brain Products, Munich, Germany), and recorded via a computer running Brain Vision Recorder software (Brain Products, Munich, Germany). The EEG was continuously sampled at 500 Hz with a bandpass of 0.016–100 Hz. Electrode recording impedances were held beneath 10 kΩ. All EEG channels were recorded against a Vertex reference (Cz) with the ground at AFz. There were 62 recording electrodes on the scalp in a 10–10 configuration and two on the earlobes. EEG were collected for 53 of the 56 participants (*n* = 2 nPTSD and *n* = 1 sPTSD not recorded).

#### Heart Rate

Heart rate (HR) in beats per minute was determined from the continuously recorded electrocardiogram (ECG). ECG was acquired using a BIOPAC ECG100C amplifier at a sampling rate of 1 kHz with a low pass filter of 35 Hz and a high pass filter of 0.5 Hz.

#### Skin Conductance Level

Skin conductance level (SCL) was recorded using a BIOPAC SCL100C amplifier and a skin resistant transducer (TSD203) from the middle phalanx of the first and ring finger of the participant’s non-dominant hand at a sampling rate of 500 Hz with a low pass filter of 1.0 Hz.

### Procedure

Eligible participants were invited for the experimental session which took part in the Washington Singer Laboratories, at the University of Exeter and received a pack with an information sheet, consent form and questionnaires (PCL-C, SCS-SF, DASS, and FSCRS). On the day of testing, after giving written informed consent, participants were taken into the lab and fitted with the recording equipment and given verbal instructions of the experimental tasks that they would be participating in.

**FIGURE 1:**
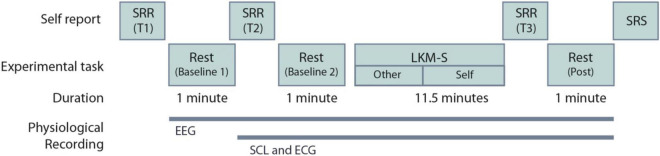
Experimental procedure for the Loving-Kindness Meditation for the self (LKM-S). The timing of the electroencephalogram (EEG), skin conductance level (SCL), and electrocardiogram (ECG) recordings are shown. SRR are self-reported visual analog scales repeated at three time points T1-3; SRS are the self-reported measures that are collected at a single time point only, these include the PCL-C, SCS-SF, DASS and FSCSR; see Section “Self-Report Measures”.

At the beginning and the end of the experimental procedure, participants were asked to complete a self-referential task using the “Me/Not-Me” response task not reported here. Participants then completed a mood state measure using self-reported visual analog scales (VAS); described in Section “Recording Devices.” One minute of resting-state EEG recording was taken with eyes closed. The VAS were then repeated. Participants then listened to a self-compassion induction delivered via headphones. The self-compassion induction is an eleven-and-a-half-minute Loving-Kindness Meditation for the self (LKM-S) developed and validated in Kirschner et al. (2019). Listeners are invited to focus compassion toward others initially (minutes 1–5) and then on to directing the same feelings of compassion toward themselves. All participants listened to the same script whilst their individual psychophysiological measurements (EEG, ECG, and SCL) were recorded. After the induction the VAS were completed for a third time followed by 1 min of resting-state EEG, ECG, and SCL were recorded with eyes closed. Finally, participants were asked to complete four self-reported measures detailed in Section “Recording Devices.” Figure 1 summarizes the experimental procedure. After the experiment participants were instructed to follow a mood neutralization task (MNT) (Nolen-Hoeksema and Morrow, 1993).

### Data Pre-processing and Analysis

#### Electroencephalogram

EEG data were re-referenced offline to linked mastoids and band pass filtered 1–40 Hz. EEG was inspected and bad channels manually removed. Two data sets were rejected (*n* = 1 fPTSD, *n* = 1 nPTSD) due to a faulty cap producing many bad channels. EEG artifacts (ocular, cardiac, and muscle) were removed by applying independent component analysis (Jung et al., 2000). Missing channels were then found by spherical interpolation.

Each 1-min segment of EEG data was divided into 4 s epochs with 50% overlap. Epochs containing significant physiological artifacts with amplitudes exceeding ±100 μV were rejected (Wahbeh and Oken, 2013; Meyer et al., 2016). Two subjects (*n* = 1 fPTSD and *n* = 1 nPTSD) were excluded as >75% of the epochs were rejected in each set. The percentages of accepted epochs did not differ between diagnostic groups; see Table 1. LKM-S minute 1 is significantly lower than at baseline B, LKM-S minute 2, LKM-S minute 3, LKM-S minute 4 and LKM-S minute 5 (*p* < 0.05). This minute is excluded from subsequent analysis.

**TABLE 1:** Mean percentages of accepted segments for each minute, resting-state baseline (B) each minute (1–11) of the audio exercise (LKM) and the post-induction resting-state baseline (P).

B	LKM											P
	1	2	3	4	5	6	7	8	9	10	11	
93%	87%	93%	94%	92%	94%	91%	91%	91%	93%	91%	88%	89%

#### Alpha Asymmetry

All epochs free of artifacts were subjected to a fast Fourier transformation using a Hamming window over the distal 50% of each epoch. By averaging segments, we derived estimates of spectral power (μV2) for 0.5-Hz bins, averaged between 8 and 13 Hz to obtain power density (μV2/Hz) in the alpha band. Our analytic strategy was to quantify activity recorded over the left and right brain regions. We therefore averaged electrode sites within the anterior regions (left: F3, F5, F7, FC5, FT7, and T7; right: F4, F6, F8, FC6, FT8, and T8) and posterior regions (left: CP5, P3, P5, and P7; right: CP6, P4, P6, and P8). This approach has the advantage of reducing the amount of data and, according to the Spearman–Brown prophecy formula, increasing reliability of brain asymmetry measures. Power density values were log-transformed using the natural log to normalize data. We computed asymmetry scores (right minus left hemisphere) for each minute of the baselines and audio.

#### Microstate Analysis

The artifact free data from the baseline (B) and LKM-S minutes were down-sampled to 125 Hz and filtered to 1–30 Hz (ref). Microstate segmentation was performed using the EEGLAB microstate toolbox (Poulsen et al., 2018). The global field power (GFP) is computed as the standard deviation over all channels at each time point. EEG topographies at the peaks of the GFP have high signal to noise ratio. Consecutive peak maps may have the same potential shape but reverse polarity (Wackermann et al., 1993). We concatenated and submitted 100 peak maps from each subject and each minute to *k*-means clustering for *k* = 2,…, 10. The optimal number of clusters was chosen to be four in line with previous studies (Khanna et al., 2015).

**FIGURE 2:**
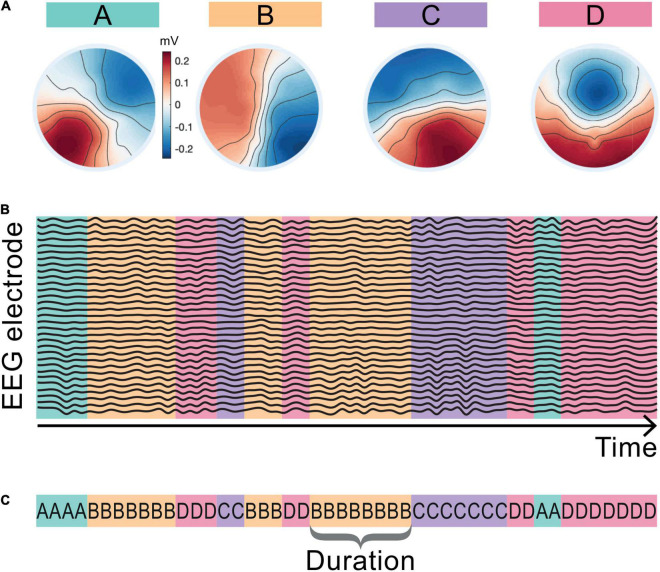
Microstate maps and back-fitting. The four EEG microstate topographies resulting from the microstate segmentation of the whole cohort including the baseline and LKM-S epochs are shown in panel **(A)**. Maps are aligned and labeled A–D in line with the four canonical microstate maps in the literature. Schematic in panel **(B)** shows assigned map that best fits the EEG topography at each sampling point of the EEG data. Panel **(C)** shows the corresponding microstate sequence and the definition of microstate duration.

Here we are interested in how the temporal dynamics of the microstate sequences change from baseline during the LKM-S. To eliminate slight variation from different group level maps and from baseline to LKM-S we chose to concatenate maps from all groups and all minutes. The resulting four microstate maps are shown in Figure 2A and are labeled in line with the four classic maps (Khanna et al., 2015; Michel and Koenig, 2018).

The maps were fitted back onto the data in a winner-takes-all manner by choosing the map with the highest correlation at each time point resulting in a microstate sequence for each minute and each subject; see the schematic representation in Figure 2B. The mean topographic variance of the EEG data explained by these four maps is 75–3% in line with commonly reported values in the literature (Khanna et al., 2015). The *duration* of a microstate is the length of time that it is active (consecutively assigned to the data) before transitioning to another microstate; a single duration of microstate B is shown in Figure 2C. Microstate sequences can be fully classified by their transition probabilities and the microstate durations (Creaser et al., 2019). For each individual we computed the mean duration spent in each microstate for the baseline and each minute of the LKM-S. To mitigate for individual variation, we computed the *duration response* by subtracting the mean duration of each microstate at baseline from the mean duration at each minute of the LKM-S. We then computed the mean and standard error of the duration response for each group in each minute of the LKM-S.

#### Source Localization

Source localization was performed on the microstate maps using the using the FieldTrip toolbox for EEG/MEG-analysis, developed at the Donders Institute for Brain, Cognition and Behavior^1^ (Oostenveld et al., 2011). The FieldTrip template head model was used (Oostenveld et al., 2003). Sources were identified both on the cortical surface and within the cortical gray matter using the eLoreta method (Pascual-Marqui, 2009), with the extended international 1,020 electrode template (Oostenveld and Praamstra, 2001). The cortical surface template used in FieldTrip was the MNI ICBM152 Average Brain Stereotaxic Registration Model (Grabner et al., 2006), with neuroanatomical labeling using Freesurfer (Dale et al., 1999), implemented in Brainstorm (Tadel et al., 2011). Here we used the unconstrained orientation of dipoles as the microstate maps were computed ignoring polarity. The standard FieldTrip fMRI template was used in for the intercortical source identification (Holmes et al., 1998). The template was parcellated based on the automated anatomical labeling atlas (Tzourio-Mazoyer et al., 2002) and restricted to gray matter cortical regions of interest only; the cerebellum and subcortical regions were removed. The singular value decomposition orientation constraint was applied. Once the activation level was computed in each voxel the values were *z*-scored.

#### Heart Rate and Heart Rate Variability

The raw ECG data were bandpass filtered using FIR between 0.5 and 35 Hz and 8,000 coefficients. Artifact detection (i.e., noisy, missing, or ectopic beats) and removal was performed using a template correlation and interpolation from the adjacent R-peaks (Berntson et al., 1990; Berntson and Stowell, 1998; Solem et al., 2006). The interpolation procedure was used for less than 5% of the ECG data. HR was determined using a semi-automatic R-wave detection algorithm implemented in the software AcqKnowledge (Version 4.2., BIOPAC Systems Inc., Goleta, CA, United States). Mean HR in beats per minute were then extracted from the R-waves. High-frequency HRV was determined from the artifact-free ECG by submitting a time series of the R-peaks to a fast Fourier transformation that calculated the power spectrum of the R–R interval variation for the frequency range between 0.15 and 0.4 Hz in a given time window (Berntson et al., 1997). High-frequency HRV values were transformed using a percent deviation from the mean (Ellis et al., 2008); we denote these values HRV. For the different experimental conditions, HRV values were determined in 1-min segments throughout the audio exercise. Four 1-min blocks were recorded and averaged for baseline prior the audio exercise.

#### Skin Conductance Level

Mean SCL, Maximum SCL values and minimum SCL values were extracted for the same time windows as described above and a range correction as recommended by Lykken et al. (1966) was applied to each data section for each participant to give a mean SCL corrected for individual differences. The formula for this was: Corrected SCL = (SCL mean − SCL min)/(SCL max − SCL min).

To obtain measures of HR, HRV and SCL change throughout the audio exercise and in order to control for individual differences we calculated participants’ response values. These response values were calculated by subtracting values the averaged baseline values from each minute of the audio exercise for each participant.

### Statistical Analyses

Prior to answering the research questions, the data were examined for missing values, normal distribution, and outliers. Data were analyzed using custom code in MATLAB and R. For the SCL response, HRV response, the response at minute one of the LKM-S was found to be non-normal and Kruskal–Wallis tests were used. At minute 7 the SCL response data is normal and a one-way ANOVA is used to compare between groups. *Post hoc* pairwise comparison tests were performed between groups with Bonferroni correction applied. Averaging the SCL and HRV response during the focus on other (min 1–5) and self (min 6–11), the SCL means were found to be normal and a repeated measures ANOVA (within = focus, between = group) was used. The HRV means were found to be non-normal during the focus-other, however, a repeated measures ANOVA (within = focus, between = group) was used due to the absence of a non-parametric version of the test. For the alpha asymmetry scores, we compute paired *t*-tests between groups at minutes 1 of the LKM. We average over two time points (others minutes 1–5; self minutes 6–11) then compute a repeated measures ANOVA (rmANOVA) (within = focus, between = group). For the microstate duration analysis, we use a repeated measures ANOVA (within = microstates B and C durations, between = group). Note that here we only performed statistical analysis on microstates B and C because we observed (based on visual inspection) that these microstates had a different duration response between groups during the LKM-S, whereas microstates A and D appear consistent across groups during the LKM-S. For each rmANOVA *post hoc* tests were performed on significant results only and Bonferroni correction applied. Zero order correlations were computed using Spearman correlation coefficient. Stepwise regression modeling (both ways, forward and backward) was performed to identify predictors for the microstate durations.

## Results

### Self-Report Measures

**FIGURE 3:**
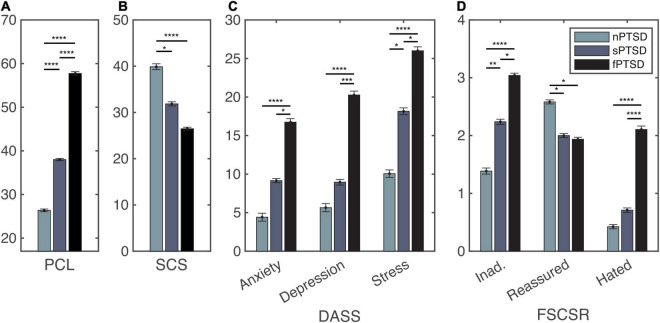
Self report measure statistics for the PTSD Checklist (PCL) panel **(A)**, self-compassion scale (SCS) panel **(B)**, depression, anxiety and stress scales (DASS) panel **(C)**, and forms of self-criticism/self-reassuring scale (FSCSR) panel **(D)**. Mean scores are shown as bars, error bars show standard error for each group nPTSD, sPTSD, fPTSD. Significant differences between groups are indicated by *p < 0.05, **p < 0.01, ***p < 0.001, ****p < 0.0001.

Following recruitment and screening a final sample size of 56 participants completed the study. Table 2 summarizes the subject characteristics and three group comparisons. Of the 56 participants, *n* = 19 (34%) satisfied the criteria for fPTSD, *n* = 20 (36%) satisfied the criteria for sPTSD and *n* = 17 (30%) satisfied the criteria for nPTSD. One individual did not meet the intrusion symptom criterion but did meet the avoidance and numbing, and hyperarousal criteria, and had a PCL-C above 44 so they were included in the sPTSD group. Comparison between groups was performed using paired *t*-tests with Bonferroni correction. Figure 3A shows the PCL-C scores for each group. The fPTSD group has the highest mean score and nPTSD group has the lowest mean score. There are significant differences (*p* < 0.0001) between all three groups. Figure 3B shows the SCS-SF scores. The nPTSD group is significantly higher than both the fPTSD (*p* < 0.0001) and sPTSD (*p* < 0.05) groups. There is no significant difference (*p* = 0.177) between the fPTSD and sPTSD groups indicating they have similar levels of self-compassion. Figure 3C shows the group level DASS scores for each subscale. In all three subscales the fPTSD group is significantly higher than the nPTSD group (*p* < 0.0001). The nPTSD and sPTSD groups are not significantly different for anxiety or depression, but sPTSD is significantly higher than nPTSD for stress (*p* < 0.05). The sPTSD group is significantly lower than the fPTSD group for the anxiety and stress subscales (*p* < 0.05) and depression subscale (*p* < 0.001). Figure 3D shows group level FSCSR measures for each subscale. For the Inadequate and Hated subscales, the fPTSD group is significantly higher than the nPTSD group (*p* < 0.0001). For the Inadequate subscale the sPTSD group is significantly higher than the nPTSD (*p* < 0.01) and lower than the fPTSD group (*p* < 0.05). For the Hated subscale the sPTSD group is significantly lower than the fPTSD group (*p* < 0.0001) but not different from the nPTSD group. On the Reassured subscale the nPTSD group is significantly higher than both the fPTSD and sPTSD groups (*p* < 0.05) whereas the full and sPTSD groups are not significantly different. These FSCSR scores are in line with the expectations that the fPTSD group has the highest inadequate and hated-self scores. They align with expectations that the nPTSD group shows the highest reassured-self scores and the scores of the sPTSD group are in between the two for each scale.

**TABLE 2:** Characteristics, numbers, and percentages for all participants and each of the three PTSD groups; no PTSD (nPTSD), full PTSD (fPTSD), and subsyndromal PTSD (sPTSD) as defined in Section “Self-Report Measures”.

	All participants *N* = 56	nPTSD *N* = 17	fPTSD *N* = 19	sPTSD *N* = 20	Test statistic (χ^2^)	df	*p*
Sex	Male = 11 (19.6%) Female = 45 (80.4%)	Male = 4 (23.5%) Female = 13 (76.5%)	Male = 4 (21%) Female = 15 (79%)	Male = 3 (15%) Female = 17 (85%)	0.45974	2	0.7946
Marital status	Single = 42 (75%) Married = 14 (25%)	Single = 11 (64.7%) Married = 6 (35.3%)	Single = 14 (73.7%) Married = 5 (26.3%)	Single = 17 (85%) Married = 3 (15%)	2.045	2	0.3597
Occupation	Employed = 15 (26.8%) Unemployed = 3 (5.4%) Student = 36 (64.3%) Retired = 2 (3.6%)	Employed = 7 (41.2%) Unemployed = 1 (5.9%) Student = 9 (52.9%) Retired = 0 (0%)	Employed = 2 (10.5%) Unemployed = 2 (10.5%) Student = 14 (73.7%) Retired = 1 (5.3%)	Employed = 6 (30%) Unemployed = 0 (0%) Student = 13 (65%) Retired = 1 (5%)	6.7713	6	0.3425
Education	Standard = 40 (71.4%) Higher = 16 (28.6%)	Standard = 9 (52.9%) Higher = 8 (47.05%)	Standard = 16 (84.2%) Higher = 3 (15.8%)	Standard = 15 (75%) Higher = 5 (15%)	4.4931	2	0.1058
Medication	No = 42 (75%) Yes = 14 (25%)	No = 15 (88.2%) Yes = 2 (11.7%)	No = 10 (52.6%) Yes = 9 (47.4%)	No = 17 (85%) Yes = 3 (15%)	7.7251	2	0.02101*
Trauma type	Accidental = 19 (33.9%) Interpersonal = 32 (57.1%) Both = 5 (8.9%)	Accidental = 8 (47.05%) Interpersonal = 8 (47.05%) Both = 1 (5.9%)	Accidental = 4 (21.1%) Interpersonal = 13 (68.4%) Both = 2 (10.5%)	Accidental = 7 (35%) Interpersonal = 11 (55%) Both = 2 (10%)	2.7973	4	0.5923
Number of traumas	Single = 42 (75%) Multiple = 14 (25%)	Single = 13 (76.5%) Multiple = 4 (23.5%)	Single = 14 (73.7%) Multiple = 5 (26.3%)	Single = 15 (75%) Multiple = 5 (15%)	0.0372	2	0.9816
Meditation	No = 39 (69.6%) Yes = 17 (30.4%)	No = 14 (82.4%) Yes = 3 (17.6%)	No = 12 (63.2%) Yes = 7 (36.8%)	No = 13 (65%) Yes = 7 (35%)	1.8809	2	0.3905
Handedness	Right = 51 (91.1%) Left = 5 (8.9%)	Right = 15 (88.2%) Left = 2 (11.8%)	Right = 18 (94.7%) Left = 1 (5.3%)	Right = 18 (90%) Left = 2 (10%)	0.5103	2	0.7748

*We show the between group chi-square test statistic, degrees of freedom (df) and p-value.*

*p < 0.05. Education: Standard = A-levels/GNVQ; Higher = Degree; Medication: Psychotropic medication (not recently changed), Trauma type: accidental trauma (considered to be an experience brought about through no purposeful intent) or interpersonal trauma [“family and intimate partner violence…violence between individuals who are unrelated…child abuse, violence, random acts of violence, rape, or sexual assault by strangers, and violence in institutional settings…sudden bereavement” (World Health Organization, 2002, p. 14)].

### Self-Compassion and Self-Criticism Response

#### Self-Compassion

**FIGURE 4:**
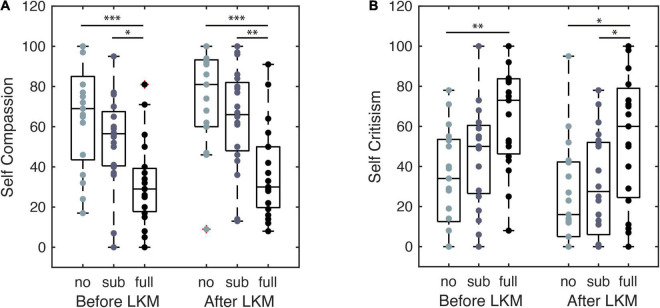
Self compassion **(A)** and self-criticism **(B)** self-report scores from before and after the LKM-S. Each group, labeled no, sub, and full PTSD, are shown at each time point with significance levels *p < 0.05, ^**^p < 0.01, ^***^p < 0.001.

The self-reported self-compassion scores recorded before and after the LKM-S are shown for each group in Figure 4. The mixed ANOVA with time (pre, post LKM-S) as within-subject factor and group (nPTSD, fPTSD, and sPTSD) as between subject factor revealed significant main effects of time *F*(1,53) = 8.713, *p* = 0.005, $\eta_{\text{p}}^{2}$ = 0.328, and group *F*(2,53) = 12.94, *p* < 0.001, $\eta_{\text{p}}^{2}$ = 0.141, and a non-significant time by group interaction [*F*(2,53) = 0.228, *p* = 0.797, $\eta_{\text{p}}^{2}$ = 0.009]. LKM-S Bonferroni adjusted *p*-value, pairwise comparisons of the main effect of self-compassion score were non-significant. Pairwise comparisons show that the nPTSD self-compassion score is significantly higher than the fPTSD before the LKM-S (*p* < 0.001) and after the LKM-S (*p* = 0.000105); and the sPTSD self-compassion score is significantly higher than the fPTSD before (*p* = 0.0162) and after (*p* = 0.00425) the LKM. There were no significant differences between the nPTSD and sPTSD groups’ self-compassion scores either before or after the LKM.

#### Self-Criticism

The self-reported self-criticism scores recorded before and after the LKM-S are shown for each group in Figure 4. The mixed ANOVA with time (pre, post LKM-S) as within-subject factor and group (nPTSD, fPTSD, and sPTSD) as between subject factor revealed significant/ns main effects of time *F*(1,53) = 17.517, *p* < 0.001, $\eta_{\text{p}}^{2}$ = 0.248, and group *F*(2,53) = 6.678, *p* = 0.003, $\eta_{\text{p}}^{2}$ = 0.201, and a non-significant time by group interaction [*F*(2,53) = 0.59, *p* = 0.558, $\eta_{\text{p}}^{2}$ = 0.022].

LKM-S Pairwise comparisons show that self-criticism score is significantly lower for the nPTSD group than the fPTSD group before the LKM-S (*p* = 0.00185) and after the LKM-S (*p* = 0.0167); and the self-criticism score is significantly lower for the sPTSD than the fPTSD after (*p* = 0.0499). The Bonferroni adjusted p-value, pairwise comparisons of the main effect of self-criticism score was non-significant.

### Skin Conductance Level, Heart Rate, and Heart Rate Variability Response

#### Skin Conductance Level

**FIGURE 5:**
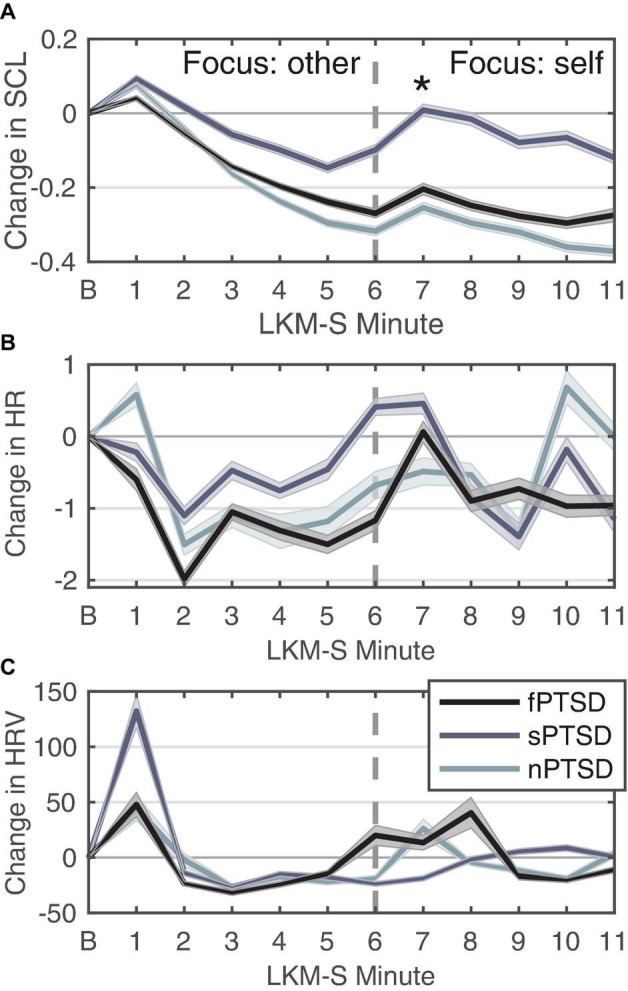
Change in SCL **(A)**, HR **(B)**, and HRV **(C)** for each group in response to each minute of the LKM-S. Error bars are ± 1 SE. Panel **(B)** is the baseline minute, resting state, eyes closed. LKM-S minutes 1–5 focus on others, minutes 6–11 focus on self. In panel **(A)** sPTSD is significantly higher than nPTSD at minute 7 *p < 0.05.

Figure 5A shows the SCL for each PTSD group. Peaks in SCL can be seen in all three groups in minutes 1 and 7 of the LKM. The SCL response at minute 1 of the LKM-S revealed no significant difference between groups (Kruskal–Wallis chi-squared = 1.1696, df = 2, *p* = 0.5572). At minute 7 the ANOVA for group was significant *F*(2) = 4.579, *p* = 0.0147, $\eta_{\text{p}}^{2}$ = 0.150. Pairwise comparison testing revealed the sPSTD is significantly higher than the nPTSD group (*p* = 0.021). Averaging the SCL response during the focus on other (min 1–5) and self (min 6–11) the repeated measures ANOVA (within = focus, between = group) revealed a significant difference in the main effects of PTSD group *F*(2,52) = 4.106, *p* = 0.0220, $\eta_{\text{p}}^{2}$ = 0.136, and LKM-S focus *F*(1,52) = 29.669, *p* < 0.001, $\eta_{\text{p}}^{2}$ = 0.363, and interaction between group and focus *F*(2,52) = 5.322, *p* = 0.008, $\eta_{\text{p}}^{2}$ = 0.170. Pairwise comparison between groups at each level show that the sPTSD group has significantly higher SCL response than the nPTSD group during the self-focus (min 6–11, *p* = 0.0105). Pairwise comparison of focus for each group revealed a significant decrease in SCL from focus on other to focus on self for the nPTSD (*p* = 0.00279) and fPTSD (*p* = 0.0383) groups only.

#### Heart Rate

Figure 5B shows the HR response for each group. The HR response at minute 1 of the LKM-S was not found to be significant [*F*(2) = 0.857, *p* = 0.43, $\eta_{\text{p}}^{2}$ = 0.0319]. Averaging the HR response during the focus on other (min 1–5) and self (min 6–11) the repeated measures ANOVA (within = focus, between = group) revealed no significant differences in the main effects of group [*F*(2,52) = 0.24, *p* = 0.786, $\eta_{\text{p}}^{2}$ = 0.009], focus [*F*(1,52) = 0.3.88, *p* = 0.054, $\eta_{\text{p}}^{2}$η^2^ = 0.069] or interaction [*F*(2,52) = 0.386, *p* = 0.682, $\eta_{\text{p}}^{2}$ = 0.015].

#### Heart Rate Variability

Figure 5C shows the HRV response for each group. Visual inspection revealed a peak at 1 min then the decrease and then another increase again after minute 5. The sPTSD group has the largest mean peak in the first minute, although this was not found to be significant (Kruskal–Wallis chi-squared = 3.5482, df = 2, *p* = 0.1696). For this group, the HRV remains lower until a peak in minute 9, this pattern during the self-focus resembles the depressed group before MBCT treatment (Kirschner et al., 2022). Averaging the HRV response during the focus on other (min 1–5) and self (min 6–11) the repeated measures ANOVA (within = focus, between = group) revealed no significant differences in the main effects of group [*F*(2,52) = 2.14, *p* = 0.128, $\eta_{\text{p}}^{2}$ = 0.076], focus [*F*(1,52) = 0.008, *p* = 0.930, $\eta_{\text{p}}^{2}$ = 0.000148], or interaction [*F*(2,52) = 0.832, *p* = 0.441, $\eta_{\text{p}}^{2}$ = 0.031].

### Alpha Asymmetry

#### Anterior

**FIGURE 6:**
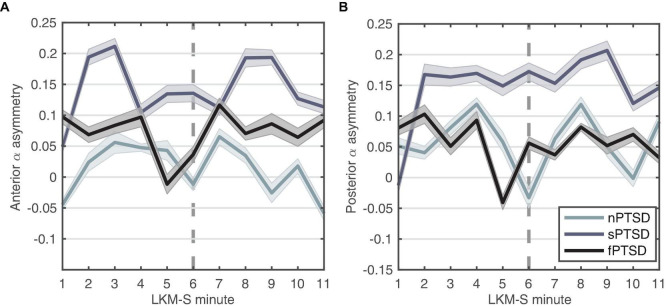
Alpha-asymmetry (R-L) computed for **(A)** anterior and **(B)** posterior during each minute of the LKM-S for each group. Error bars are ±1 SE. In minutes 1–5 participants focus on others, minutes 6–11 participants focus on the self.

We hypothesize that large positive anterior alpha asymmetry scores indicate exaggerated withdrawal motivation characteristic of anxiety disorders. Figure 6A shows that the anterior alpha asymmetry score for the f- and sPTSD groups are larger than the nPTSD, inspection of the power in each hemisphere reveals that this is due to an increase in alpha power in right hemisphere in both groups. The repeated measures ANOVA (within = focus, between = group) revealed no significant differences in the main effects of group [*F*(2,46) = 2.19, *p* = 0.123, $\eta_{\text{p}}^{2}$ = 0.087], focus [*F*(1,46) = 0.01, *p* = 0.916, $\eta_{\text{p}}^{2}$ = 0.000246], or interaction [*F*(2,46) = 0.61, *p* = 0.547, $\eta_{\text{p}}^{2}$ = 0.026].

#### Posterior

We hypothesize that the magnitude of the parietal alpha asymmetry will distinguish between the groups. Figure 6B shows the posterior alpha asymmetry scores. The sPTSD group appears consistently higher than the n- and f-PTSD groups, and that this is driven by both an increase in right and decrease in left hemisphere alpha power. The repeated measures ANOVA (within = focus, between = group) revealed no significant differences in the main effects of group [*F*(2,46) = 1.524, *p* = 0.229, $\eta_{\text{p}}^{2}$ = 0.062], focus [*F*(1,46) = 0.121, *p* = 0.729, $\eta_{\text{p}}^{2}$ = 0.003], or interaction [*F*(2,46) = 1.987, *p* = 0.149, $\eta_{\text{p}}^{2}$ = 0.08]. Note that the homogeneity of variance assumption is violated for this posterior data.

### Microstate Duration

**FIGURE 7:**
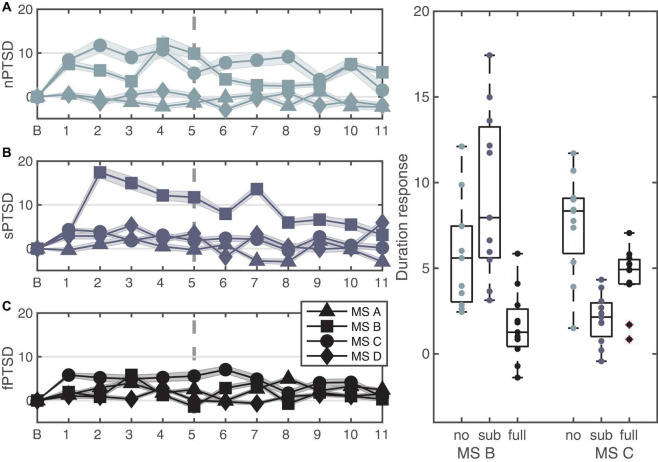
Time series of the LKM-S showing the duration response from baseline B of each microstate for each group nPTSD **(A)**, sPTSD **(B)**, and fPTSD **(C)**. The line at minute 6 indicates the shift in focus from others (1–5) to self (6–11). Panel **(D)** shows significant differences in duration response over the whole LKM-S between groups for microstates MS B and MS C; *p < 0.05, **p < 0.01, ****p < 0.0001.

Figures 7A–C shows the change in duration for the four microstates for each group. We performed a repeated measures ANOVA where between is group and within is microstate (MS B and C). The data satisfy normality assumptions and there are no outliers, however, violate homogeneity of variances and covariances assumptions. The rmANOVA gave a statistically significant simple main effect of group *F*(2,30) = 7.401, *p* = 0.002, $\eta_{\text{p}}^{2}$ = 0.330, a non-significant main effect of MS *F*(1,30) = 1.870, *p* = 0.18, $\eta_{\text{p}}^{2}$ = 0.059 and a significant interaction between PTSD group and MS *F*(2,30) = 24.657, *p* < 0.0001, $\eta_{\text{p}}^{2}$ = 0.622. Pairwise comparisons with Bonferroni correction show that the mean change in durations for MS B is significantly lower for the fPTSD compared to the nPTSD group (*p* = 0.0289) and compared to sPTSD (*p* < 0.0001) but nPTSD and sPTSD are not significantly different (*p* = 081). Pairwise comparisons with Bonferroni correction show that the mean change in durations for MS C for the nPTSD group is significantly higher than for the fPTSD group (*p* = 0.00743) and for the sPTSD group (*p* < 0.0001). The duration response for the fPTSD group is also significantly higher than the sPTSD group (*p* = 0.0393). Pairwise comparisons with Bonferroni correction of the duration response between microstates revealed no significant difference between MS B and C for nPTSD group (*p* = 0.197), but MS C is significantly higher than MS B for the fPTSD group (*p* = 0.00238) and MS B is significantly higher than MS C for the sPTSD group (*p* < 0.001). The results of the significance comparison are shown in Figure 7D.

### Source Localization of Microstates

**FIGURE 8:**
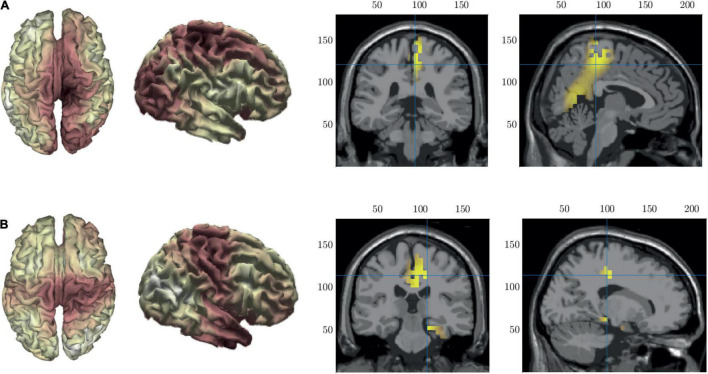
Source localization of microstate maps B row **(A)** and C row **(B)** shown in Figure 3. The first two columns show the source localization on the cortical surface. The last two columns show the sources (*z*-score > 2) identified using the template fMRI head model.

To help with the interpretation of the changes in duration we performed source localization on microstates B and C to identify the key generators of each spatial pattern. The results of the source localization are shown in Figure 8 and Table 3. All cortical regions that had >8 active voxels identified are listed in Table 3 for each microstate. Figure 8A shows the results of the source localization for MS B. The largest regions of activation (highest number of voxels with *z*-score > 2) are in the parietal and occipital regions, specifically the precuneus, median cingulate cortex and the primary visual cortex (right lingual). The regions with the highest levels of activation (largest *z*-score) are in the frontal lobe, the paracentral lobe and supplementary motor area. Figure 8B shows the results of the source localization for MS C. The largest regions of activation are the frontal and temporal regions, specifically the median cingulate cortex and the parahippocampal and fusiform gyri. The parahippocampal gyrus has the highest levels of activation.

**TABLE 3:** List of cortical regions identified via source localization for microstate maps B and C.

Source localization of MS B		Source localization of MS C	
# voxels (*z*-score > 2)	Cortical region	# voxels (*z*-score > 2)	Cortical region
44	R precuneus (parietal)	41	R Median cingulate and paracingulate gyri
43	R Lingual gyrus (occipital)	36 (3)	R Parahippocampal gyrus (temporal)
37	R Calcarine fissure and surrounding cortex (occipital)	34	R Fusiform gyrus (temporal)
32 (1)	R Median cingulate and paracingulate gyri	19 (6)	L Median cingulate and paracingulate gyri
22 (6)	R paracentral lobule (frontal lobe)	15 (1)	R supplementary motor area (frontal lobe)
20 (3)	R supplementary motor area (frontal lobe)	16	Bilateral paracentral lobule
14	R posterior cingulate gyrus		

*The number of active voxels in each region is given and in brackets the subset of those that had a z-score > 2, if any. Note that only the regions that contained >8 identified voxels are listed. R, right; L, left; MS, microstate.*

### Correlations of Neural and Physiological Measures

Mean posterior alpha asymmetry score over the whole LKM-S was found to be positively, though not significantly, correlated with LKM-S-related change in self-compassion *r*(47) = 0.26, *p* = 0.075. Mean anterior alpha asymmetry score over the whole LKM-S was found to be positively correlated with LKM-S-related change in self-criticism *r*(47) = 0.29, *p* = 0.044, although this was not significant after Bonferroni correction.

We performed stepwise regression with microstate MS B as outcome variable. The following predictors were submitted based on results above: PCL total score, SCL at minute 7, mean SCL response during focus-other and mean during focus-self, mean over whole LKM-S for the HRV response, and anterior and posterior alpha asymmetries. Results of the multiple linear regression indicated that the overall model was significant for MS B, [*F*(3,44) = 11.01, *p* < 0.0001] and explained 39% of variance. The individual significant predictors were examined further and found that PCL total was negatively correlated with MS B (*b* = −0.16, *SE* = 0.08, *t* = −2.05, *p* = 0.047); anterior alpha asymmetry (*b* = 19.37, *SE* = 9.89, *t* = 1.96, *p* = 0.057); and posterior alpha asymmetry (*b* = 16.87, *SE* = 9.92, *t* = 1.70, *p* = 0.096) were positively correlated with MS B.

## Discussion

The aim of this study was to establish brain and psychophysiological responses to a brief single session self-compassion cultivation in trauma survivors with full PTSD, subthreshold PTSD and without PTSD. We analyzed the response of these individuals to a validated self-compassion exercise using EEG alpha asymmetry and novel EEG microstate analysis and complemented these with psychophysiological (skin conductance, heart rate, and heart rate variability) measures and self-report measures of self-compassion and self-criticism.

### Physiological Response to Self-Compassion Cultivation

Previous research has proposed that the threat response to the LKM-S can been used as a proxy for excessive self-criticism and rumination (Kirschner et al., 2022). In individuals with depression this manifests as an adverse physical arousal response to the LKM-S indicated by significantly elevated skin conductance levels (Kirschner et al., 2022). SCL is a well-known physiological measure of activation of the sympathetic nervous system and defense response. To our knowledge there have not been any trails looking at the response to the LKM-S in individuals with PTSD.

Our results showed that the sPTSD group has significantly higher skin conductance level (SCL) response than the nPTSD group during the self-focus portion of the LKM-S. Visual comparison to the LKM-S response for people with remitted depression (Kirschner et al., 2022) revealed that the sPTSD response was similar to the remitted depressed group response, where the values of the SCL for the depressed group remain higher than the healthy control. We found the nPTSD and fPTSD SCL response are not significantly different, and both appear similar to the control group in Kirschner et al. (2022) and the healthy sample in Kirschner et al. (2019). We do not control for depression in this study. There is a well-known complex interaction between the physiological and neurological mechanisms of depression, anxiety and PTSD (Kemp et al., 2010; Zerna et al., 2021). Moreover, fPTSD, sPTSD, and nPTSD significantly differ in DASS depression scores, which makes statistically controlling for depression problematic in this study (Miller and Chapman, 2001). Here we are interested in the transdiagnostic mechanism of negative-self and we show that the physiological response to the LKM can differentiate between subtypes of PTSD. The LKM response may, therefore have the potential to differentiate PTSD and depression. This would require a dedicated study, with a larger sample size than used here, which is left to future work.

Our results indicate that the sPTSD group experience higher levels of stress and elevated defense in response to the LKM. This partially supports our hypothesis of a higher threat response in the subthreshold PTSD group in reaction to the shift of focus to the self. This response of higher arousal levels, and lower emotional numbing levels, as measured by the PCL-C, in this group, may contribute to the elevated threat response. Individuals with high levels of avoidance caused by overactivation of the withdrawal system as indicated by high skin conductance and lower heart rate variability, are associated with high attrition rates and poor effectiveness of treatment (Boyd et al., 2017). Griffin et al. (1997) have shown that high arousal levels may indicate good treatment response. The use of the LKM-S response may facilitate stratification of individuals to treatment plans in the clinic.

### Neural Response to Self-Compassion Cultivation

This is the first study that considers the alpha asymmetry response to cultivating self-compassion with LKM-S. Characteristic patterns of alpha asymmetry have been associated with PTSD during trauma related tasks, notably higher right sided activation (Metzger et al., 2004; Rabe et al., 2006; Kemp et al., 2010). Increased alpha activity in areas measured by Pz-P4 were found to differentiate individuals with PTSD from controls (Imperatori et al., 2014). We found that although there was a trend for the sPTSD group to have higher posterior alpha asymmetry during the LKM-S than the n- and fPTSD groups, this was not found to be significant. Our results did not support our hypothesis that the magnitude of the parietal alpha asymmetry would be greater in the sPTSD group. This indicates the potential of alpha asymmetry as a neural correlate of self-compassion, but here we are limited by the statistical power of the study which is not suitable to detect small effect sizes. The lack of significance in this and other studies may indicate that alpha asymmetry is not sensitive enough to be used as a neural biomarker for PTSD.

To address the limitations of alpha asymmetry we complement our study with EEG microstate analysis. EEG microstates have been shown to be sensitive to transient changes in brain function and so are a suitable tool for measuring changes in mentation in response to the LKM-S intervention. One advantage of the microstates approach is the evaluation of the both spatial and temporal dynamics of brain activity. We find four microstates over the whole cohort that align with the four classic resting state microstate topographies (denoted A, B, C, and D) (Khanna et al., 2015; Michel and Koenig, 2018). This shows consistency of both the LKM and resting states maps with previous studies and internal consistency between the groups before and during the LKM. The microstate approach then allowed us to examine the temporal activity in response to the LKM to distinguish PTSD in trauma survivors. A further advantage of the microstates approach is the association of the classic microstate maps with underlying brain networks, including saliency and attention networks (Britz et al., 2010; Yuan et al., 2012).

The EEG signal that defines each microstate topography is thought to come from the large scale coordinated activity of neurons on the cortical surface (Khanna et al., 2015). Multiple neuronal assemblies from distinct cortical regions activate synchronously, forming a network of sources that generate a certain microstate topography. Switching between different microstates can thus be interpreted as the sequential activation of different networks of neurons. The average lifespan of a microstate (mean duration) represents the stability of the underlying neural sources where longer durations indicate more stable ensembles. In task-oriented activity, measured by evoked potential response (ERP) individual microstates have been shown to influence how a stimulus is processed and reacted to. For example, in a perceptual rivalry task the stimulus that is detected depends on the active microstate at the moment the stimuli are presented (Britz et al., 2014).

We found that the durations of microstates B (MS B) and C (MS C) increase during the LKM in the nPTSD group and that this increase is sustained over many minutes. This is in line with findings that for healthy individuals duration of microstate B was found to increase during meditation (Faber et al., 2005). This indicates that B and C are the microstates that optimally process and react to the information being received (here the LKM-S).

We found that the fPTSD group had significantly shorter durations than the nPTSD group in both MS B and MS C. PCL score was identified as a predictor of MS B duration with a negative correlation, so the higher the PCL score the lower the MS B duration. Al Zoubi et al. (2019) compare microstate durations between a population with mood and anxiety disorders, and healthy controls (Al Zoubi et al., 2019). They do not distinguish between disorders or specify comorbidity. They identify a trend toward the increase of the duration of microstate C in the resting state dynamics of the mood and anxiety disorder group. They associate this with higher levels of self-referential processes in DMN that are related to depression. This is in direct contrast to the resting state microstate dynamics of a cohort of depressive patients who showed that the microstate durations, over all microstates, was significantly shorter in the depressives group (Strik et al., 1995). Here we find that the f-PTSD group, with significantly higher levels of depression, shown significantly shorter durations than the nPTSD and sPTSD groups in microstate B and C in response to the LKM-S. However, the sPTSD group also had significantly shorter durations of microstate C than the nPTSd group where there is no difference in level of depression. The discrepancy between studies, and individual microstates, indicates that depression may manifest in the brain dynamics differentially depending on whether it is the primary diagnosis or present as part of another mood or anxiety disorder, or fully comorbid. Further studies that control for depression in a variety of disorders are required.

The durations of MS C are shorter for the sPTSD group. This supports our hypothesis that there will be lower microstate durations during the LKM-S in the full and subthreshold PTSD groups than the no PTSD group. We find that the duration of MS C was lowest in sPTSD and highest in fPTSD. Duration and occurrence of microstates are modulated by vigilance level and MS C in particular is positively associated with vigilance (Krylova et al., 2020). This indicates, counterintuitively, that those with higher hyperarousal scores, that also show a higher threat response, have lower vigilance levels. This could indicate the presence of PTSD with dissociation (PTSD-DS), which is recognized as a distinct subtype of PTSD in the DSM-5 (American Psychiatric Association [APA], 2013). Here, due to the historical nature of the data the DSM-IV (The American Psychiatric Association [APA], 1994) was used during the recruitment process and so this subtype was not identified in our cohort. PTSD-DS is characterized by self-reported “zoning out” and no detectable autonomic response (Lanius et al., 2002, 2006; Boyd et al., 2017). This subtype is usually linked with higher PTSD symptom severity and detachment rather than hypervigilance (Thome et al., 2020). The PTSD-DS subtype has recently been found to be differentiated from PTSD and healthy controls by the activation patterns of underlying functional connectivity networks (Nicholson et al., 2020). Future studies should investigate the propensity for differences in neural dynamics between PTSD and PTSD-DS to be detected and differentiated by the microstate dynamics.

We note here that there are many choices of differentiating trauma survivors into distinct subgroups. Here we differentiate by PCL-C score in line with other studies that consider high, medium and low overall symptom levels (Rabe et al., 2008; Sripada et al., 2018). Applying the microstate methodology to alternatively divided subgroups, based on for example grouped symptom clusters such as levels of disassociation or depression with, for example, hyperarousal or avoidance could lead to insights into the mechanisms underlying comorbidities and identify further clinically relevant subtypes. We also note here that participants recruited for this study experienced a range of trauma types. The range of traumas indicate our results may be robust to many different types of trauma and we could expect them to generalize to, for example, combat veterans.

The microstate maps found in this study very closely resemble the classic four resting state microstate maps. We performed source localization of MS B that revealed sources in the precuneus and the primary visual cortex. In healthy individuals these areas have been associated with visuo-spatial imagery, memory retrieval and experience of agency (Kondo et al., 2005; Cavanna and Trimble, 2006). These findings agree with resting state findings for MS B which is typically associated with visual imagery (Lehmann et al., 1998). In a joint resting states EEG-fMRI study MS B was associated with bilateral activation of the occipital areas and the visual and attention resting state networks (Britz et al., 2010). The shorter durations of microstate B in the fPTSD group indicate that these underlying neural ensembles deactivate prematurely, and the associated functionality is impaired in this group. This is in line with behavior findings of impaired memory and self-experiential processing in individuals with PTSD (Thompson and Waltz, 2010).

Microstate C sources were found in the parahippocampal and fusiform gyri. The parahippocampal gyrus is associated with the spatial configuration of objects and memory encoding and retrieval (Bohbot et al., 2015). The fusiform gyrus is implicated in recognition of the self and others, facial recognition and body perception (Weiner and Zilles, 2016). These areas were also identified in a study of microstate source activation during visual concrete thought in healthy individuals (Lehmann et al., 2010). In resting state, MS C has been associated with cognitive control processes and was associated with activity in the fronto-insular cortex that is part of the saliency network (Britz et al., 2010). We find shorter durations in MS C in both SPTSD and fPTSD that indicate impairment in the neural networks responsible for the recognition of the self and others as well as impaired attentional mechanisms (saliency and vigilance).

There is some discussion around the issue of interpretation of microstate analyses. There are discrepancies in the underlying brain sources identified with the four classic microstates, mainly due to differences in methodology. A prevailing view is that it is the interplay between all four microstates that is necessary for processing thoughts and stimuli (Milz et al., 2016). This may indicate that there are various simultaneously active sources causing overlap of states that is lost due to the winner-takes-all assignment of the maps to the EEG and confounds the interpretation (Custo et al., 2017).

Our study is limited by the lack of concurrent fMRI recordings which would allow more precise identification of the brain regions activated during the LKM-S and microstate activations. Several other limitations warrant a mention here. We use self-assessment for the measures of state self-compassion, self-criticism and PTSD which are well-known to have inherent bias (Orne, 1962). Clinical interview could be used to overcome this in further studies. We have a mix of genders and it has been shown that there are gendered differences in response to trauma and so they may also be differences in response to LKM-S (Kimerling et al., 2002; Butt et al., 2019).

### Theoretical and Clinical Implications

The aim of this study was to identify neurological responses to a validated self-compassion exercise in trauma exposed individuals that could distinguish full, subthreshold and absence of PTSD. The emergence of subsyndromal PTSD as a clinically significant category (Marshall et al., 2001) has contributed to the refinement and subcategorisation of PTSD types in the DSM-5. Here we split our cohort into three groups and identify the subsyndromal category in line with our previous studies (Rabe et al., 2006, 2008). We note that a limitation of the study is that participants were included and excluded under the DMS-IV criteria. We use the PCL to differentiate the groups and so we would not expect the groups to differ if the DSM-V were used for participant selection. For example, a study of veterans has identified three (mild, moderate, and severe) latent classes of symptom severity (Contractor et al., 2015). We note that there are other ways to subdivide cohorts of trauma survivors, for example four categories of trauma survivors were identified, along the lines of the previous classification but with the addition that the moderate group was split into high re-experiencing and high numbing groups (Sripada et al., 2018). This indicates a move toward considering PTSD dimensionally, from normal to abnormal as outlined by Research Domain Criteria (RDoC) project (Insel et al., 2010). A better understanding of the pathophysiology of PTSD is also required for suitable characterization of PTSD in this spectral manner (Lobo et al., 2015). Spectral disorders are more difficult to diagnose and treat. Currently PTSD is diagnosed with self-reported questionnaires or clinical interviews and so there is a need for quantitative biomarkers to aid diagnosis. Development of neural biomarkers for PTSD is crucial for early detection (Lobo et al., 2015).

The findings presented here for microstate duration could be considered as candidate neural biomarkers for PTSD subtypes. Specifically, the change in durations of MS B and C in response to the LKM-S. Shorter durations of MS C stratify nPTSD from the s- and fPTSD groups and shorter duration of MS B stratify sPTSD from fPTSD. Moreover, duration response of microstate B is positively correlated with PCL score. This indicates that MS B duration could potentially be used to diagnose PTSD. Electroencephalogram is a popular imaging modality among researchers that has significant value in the clinic (Butt et al., 2019). EEG microstate can be reliably computed with as few as 8 electrodes which could allow for the ready translation of these findings to the clinic. Future work includes testing the use of these promising candidates for improved categorization of PTSD severity.

Another key area for future work is to extend this study to identify if and how microstate durations in response to LKM-S change after treatment. This would enable development of neural biomarkers for PTSD treatment stratification and personalization. Clinicians have a variety of treatment options available but clear delineation of who will respond to which treatment has not been identified (Watts et al., 2013). Heterogeneity in symptomology, including the increasing evidence of subtypes, has been shown to correspond to different responses to treatment (Barawi et al., 2020; Dewar et al., 2020). Individuals with PTSD that have high numbing/avoidance scores perform significantly worse, in terms of symptom reduction (Korte et al., 2016). This heterogeneity of response and identification of that subthreshold category indicate that a better understanding of the etiology and treatment of PTSD is required (Lobo et al., 2015). Here we found that posterior alpha asymmetry was positively correlated with change in state self-compassion due to the LKM, and anterior alpha asymmetry was positively correlated with change in state self-criticism. This indicates the higher the posterior asymmetry the larger the increase in self-compassion and larger the decrease in self-criticism. The higher alpha asymmetry scores are associated with the sPTSD group. This suggests that high levels of arousal, or low levels of numbing, characteristic of the sPTSD group, may indicate more responsiveness to treatment. Although, our findings were non-significant as the study was not powered for small effect sizes.

The CBT has been shown to redress the level of alpha-asymmetry in PTSD described above (Rabe et al., 2008). MBCT has been shown to bring the threat response to the LKM-S (measured by SCL and HRV) of depressed individuals closer to controls (Kirschner et al., 2022). Moreover, as alpha asymmetry level was found to be a significant predictor of the level of duration response of MS B, we hypothesize that MS B durations could be a predictor for how well individuals with PTSD respond to treatment. It remains for future work to show if (M)CBT redresses the differences in microstate duration and if this can be a predictor for treatment response.

## Conclusion

This is the first study to consider neurological responses to a validated self-compassion exercise in trauma exposed individuals. We use microstate analysis to identify differences in microstate duration that are capable of stratifying individuals with no, subthreshold and full PTSD. We relate these changes to known measure of alpha asymmetry and the symptom severity. Our findings constitute a neural biomarker for PTSD that could aid diagnosis and treatment stratification to improve the lives of trauma exposed individuals.

## Data Availability Statement

The raw data cannot be shared because the participants did not agree that their data can be shared at the consent stage. Requests to access the datasets should be directed to JLC, J.Creaser@exeter.ac.uk.

## Ethics Statement

The studies involving human participants were reviewed and approved by University of Exeter, School of Psychology Ethics Committee, local trusts, and the National Health Service National Research Ethics Service (14/SW/0055). The patients/participants provided their written informed consent to participate in this study.

## Author Contributions

JLC, JS, and AK conceptualized the study. JS collected the data. JLC and AK formal analyzed and investigated. JLC created the figures and wrote the first draft of the manuscript. JLC and AK reviewed and edited manuscript. All authors reviewed and approved the final manuscript.

## Conflict of Interest

The authors declare that the research was conducted in the absence of any commercial or financial relationships that could be construed as a potential conflict of interest.

## Publisher’s Note

All claims expressed in this article are solely those of the authors and do not necessarily represent those of their affiliated organizations, or those of the publisher, the editors and the reviewers. Any product that may be evaluated in this article, or claim that may be made by its manufacturer, is not guaranteed or endorsed by the publisher.

## Footnotes

1
http://fieldtriptoolbox.org
